# Efficacy and Tolerability of Ich Nieu Khang Dietary Supplement for Overactive Bladder

**DOI:** 10.1089/jmf.2022.0085

**Published:** 2023-04-14

**Authors:** Hoan M. Vu, Van T. H. Tran, Huy Q. Hoang, Bo Han, Ba X. Hoang

**Affiliations:** ^1^ Geriatric Department, Hanoi Hospital of Traditional Medicine, Hanoi, Vietnam.; ^2^ Department of Traditional Medicine, Hanoi Medical University, Hanoi, Vietnam.; ^3^ Natural Health Medical Center, Torrance, California, USA.; ^4^ Nimni Cordoba Tissue Engineering and Drug Discovery Laboratory, Department of Surgery, University of Southern California, Los Angeles, California, USA.

**Keywords:** food supplement, incontinence, nocturia, OAB, overactive bladder, phytotherapy

## Abstract

This study aims to assess the effectiveness and safety of plant-derived food supplement Ich Nieu Khang (INK) as a dietary supplement for overactive bladder (OAB) symptoms. A total of 50 patients 18–80 years of age with the diagnosis and symptoms of the OAB were enrolled in the study and followed up for 30 days. The INK treatment efficacy, in terms of changes in nocturnal and day-time urination frequency, urination incontinence episodes, level of OAB symptoms according to Homma's OABSS scale, sleep quality according to Pittsburg Sleep Quality Index (PSQI), and possible side effects of the INK phytotherapy, was evaluated. INK significantly improved all OAB symptoms scores with a reduction of average nocturia from 4.06 ± 1.53 to 1.14 ± 0.94, the daily average urination urgency from 7.67 ± 5.00 to 5. 82 ± 3.70, the daily average frequency of urination from 9.96 ± 4.04 to 8.00 ± 3.70, weekly average incontinence of urination from 0.92 ± 1.56 to 0.60 ± 1.02, and OABSS Homma's score decreased from 9.31 ± 1.44 to 6.8 ± 2.21. INK phytotherapy also resulted in sleep quality improvement by PSQI score decreasing from 13.11 ± 1.33 to 10.54 ± 2.21. There were no adverse effects and abnormalities in paraclinical parameters with INK therapy. The results of our study suggest that INK dietary supplement is effective and safe phytotherapy for patients with primary OAB symptoms within 30 days of treatment. Larger control clinical trials are warranted to confirm our findings and promote wider use of INK for OAB and possible other age-related urination disorders.

## INTRODUCTION

Overactive bladder (OAB) is defined as a syndrome characterized by urinary urgency, with or without incontinence, usually with increased urinary frequency and nocturia in the absence of infection or other obvious diseases. The prevalence of OAB is estimated from 10% to 20% and increases with age.^1–4^

Although OAB is not life threatening, it greatly affects the quality of life, labor productivity, and financial burden for patients and society.^5,6^ Other problems with OAB include skin ulceration and urinary tract infections. The associations between psychological conditions and OAB further complicate the health and life of affected individuals with OAB. The most frequent comorbidities are depression and anxiety. In this context, OAB deeply affects self-esteem, sexuality, and relationships.^7,8^ OAB can affect children and young adults; however, this condition is most common in patients over 40 years of age.^3^

Conventional treatment for OAB included nonpharmacological and pharmacological methods. Nonpharmacological therapy aims to educate patients about OAB and help them develop strategies to manage urge and urge incontinence.^9,10^

Pharmacological treatment for OAB mainly uses anticholinergic (also called antimuscarinic) drugs. The intended result of these drugs is to achieve some relaxation of the detrusor muscle and, consequently, improve patient symptoms.^1,11^ A systematic analysis indicated that rates of clinical improvement in urgency incontinence of antimuscarinics were greater with drugs than with a placebo. However, drugs resulted in treatment discontinuation in many cases due to troublesome adverse effects.^12^ Dry mouth and constipation are the most common side effects of antimuscarinic agents. Constipation may potentiate OAB symptoms because of excessive stool in the rectal ampulla, leading to medication discontinuation in up to 50% of patients.^13,14^ Other common adverse effects include blurred vision and drowsiness. The more serious ones include confusion, cognitive, and cardiac effects, specifically QT interval prolongation.^15,16^ As shown in a 2011 systematic review of 149 studies, treatment discontinuation rates ranged from 43% to 83% in the first 30 days, and more than half of patients never refilled the initial prescription.^11,17^

Some second-line treatment options might be considered when antimuscarinic drugs are unsuccessful. These include botulinum toxin injections directly into the detrusor muscle. In a study of 100 OAB cases, the efficacy and safety of Botox injections in the detrusor muscle were evaluated. After 4–12 weeks, 88% of patients showed improvement in bladder function. However, the effects of this treatment began to diminish after 6–9 months, and repeated treatments were necessary. In a prospective cohort study, the improvement was maintained after multiple injections, although the dropout rate after two injections was 37%.^18,19^

Surgery to augment the size of the bladder, referred to as a cystoplasty, by adding to its intraluminal surface area with the interposition of a 10–15-cm loop of the small bowel or stomach, showed efficacy in some patients. However, these are expensive procedures requiring prolonged convalescence. The benefit of reducing urination urgency may be complicated by incomplete emptying of the new bladder, which may necessitate catheterization permanently. Additionally, bowel problems may also appear after augmentation cystoplasty.^20–22^

Herbal treatments are an increasingly popular alternative for treating OAB. A survey of U.S. adults ≥18 years of age conducted by the Centers for Disease Control and Prevention indicated that 74.6% of those with OAB had used some form of phytotherapy.^23^ In the present study, we have investigated the efficacy and tolerability of Ich Nieu Khang (INK) phytotherapy in 50 patients with OAB symptoms.

## MATERIALS AND METHODS

### Research material

INK dietary supplement tablets were manufactured by Medistar Vietnam Co., Ltd. Factory and distributed by FOBIC Pharma Co., Ltd.—The Food Safety Department of the Vietnamese Health Ministry issued the Certificate of Conformity with Food Safety Regulations No. 5768/2018/DKSP. Acute and chronic toxicity tests of INK on animals were conducted at the Department of Pharmacology, Hanoi Medical University. INK was certified as a safe food supplement (Certification of Conformity [Quacert] No. 3269/19/QC-PTN). The investigated INK production lot was 010919, the production date was September 16, 2019, and the expiration date was September 15, 2022 (Table 1).

**Table 1.** Content and Composition of Ich Nieu Khang

Content	Composition, mg
*Eucommia ulmoides* herbal extract (DER: 16:1)	350
L-Carnitine fumarate	150
GO-LESS (Content Pumpkin seed extract 87.5% (EFLA^®^940) and Soy isoflavone 12.5% [Soylife^®^40])	25
Excipients: Starch, magnesium stearate, talc, PVP K30, microcrystalline cellulose, HPMC	175

### Research subjects

#### Criteria for patient selection

Patients from 18 to 80 years of age, regardless of gender, with primary OAB presented with the following symptoms, including nocturia (this is a mandatory symptom), sudden urge to urinate that requires immediate urination, and difficulty controlling urination such as difficulty holding urine and urinary incontinence.

#### Criteria of patient exclusion

Patients were excluded from study groups if they were pregnant or breastfeeding, had neurogenic OAB syndrome, with causative diseases such as urination stones, bladder tumors, stroke, heart failure, mental illnesses, prostate diseases, kidney failure, or liver failure. Patients who were taking conventional drugs/traditional medicines/food supplements or other for OAB or patients who could not adhere to treatment were also excluded.

A total of 50 patients participated in the study and were eligible for clinical evaluation.

As shown in Table 2, elderly patients (>55 years old) accounted for the highest ratios among the study subjects (76%), followed by middle-aged patients (36–55 years of age), accounting for 22%. Young subjects (18–35 years of age) accounted for the lowest ratio among study subjects (2%). The mean age of the patients in the study was 62.37 ± 10.82. Most of the patients in the study were female, accounting for 94%, and males accounted for a low ratio of 6%. The group of patients with normal body weight accounted for the highest ratio of the study subjects (68%), followed by the obese subjects (26%), and the lowest was among the thin subjects (6%). The average body mass index of the studied subjects was 16.21 ± 1.34 with the group of thin patients, 20.89 ± 1.10 with the group of normal patients, and 25.04 ± 2.51 with the group of fat patients.

**Table 2.** Demographic Characteristics of the Patients

Age group	Quantity (*N*)	Ratio (%)	The average age(X ± SD)
18–35	1	2	62.37 ± 10.82
36–55	11	22	
56–80	38	76	

BMI	Quantity (*N*)	Ratio (%)	Average BMI (X ± SD)
Thin	3	6	16.21 ± 1.34
Normal	34	68	20.89 ± 1.10
Fat	13	26	25.04 ± 2.51

Duration of illness, years	Quantity (*N*)	Ratio (%)	Average duration (X ± SD)
<1	6	12	4.46 ± 3.8 9
1–3	9	18	
3–10	29	58	
>10	6	12	

BMI, body mass index; SD, standard deviation.

The group of patients with disease duration from 3 years to under 10 years accounted for the highest ratio of the studied subjects (58%), followed by those with disease duration from 1 year to <3 years ratio of 18%, the lowest is those with <1 year of disease and from 10 years or more, accounting for 12%. The average duration of disease of the patients was 4.46 ± 3.89 years, of which the longest time was 20 years, and the shortest was 3 months (Table 2).

### Patient's medical history

The patients in the study with a history of osteoarthritis were 32 people, accounting for 64%; with a history of hypertension were 16 people, accounting for 32%; with a history of urinary infection were 16 people, accounting for 32%; and with a history of type 2 diabetes in 13 people, accounting for 26%. All patients have symptoms of urination urgency and nocturia. Urinary frequency (≥8 times from waking to bedtime) was 78%, and urinary incontinence was found in 34% of patients (Table 3).

**Table 3.** Patient's Premedical Conditions and Common Urinary Symptoms Before Treatment

	Quantity (*N*)	Ratio (%)
Hypertension		
Type II diabetes	13	26
Urinary tract infection	16	32
Osteoarthritis	32	64
Urinary urgency	50	100
Nocturia	50	100
Frequent urination	39	78
Urinary incontinence	17	34

### Research Method

#### Research design

The study was interventional and conducted by investigating the patients' clinical status before and after treatment with INK.

### Treatment protocol

Patients took INK for the first 10 days at the dose of 6 tablets/day, divided into 2 doses (3 tablets each time). From day 11 to day 30, the dose dropped to 4 tablets/day, divided into 2 doses (2 tablets each time) with warm water in 1 hour after meals.

### Research flow chart


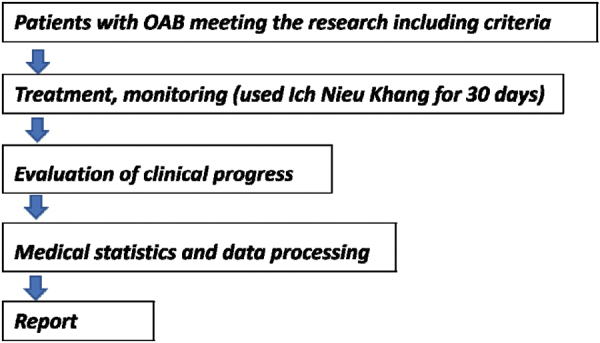


### Clinical monitoring at three time points

The clinical monitoring at three time points were before treatment (D0), after 10 days (D10), after 20 days (D20), and after 30 days (D30) of the INK treatment. The parameters for monitoring included vital function (pulse, blood pressure, respiration rate), the number of times of urination during the day, the number of times of urination at night, and the number of urinary incontinence. Urination diary was also adapted to record the time to urinate and estimate the amount of urine each time, the time of urination/urinary incontinence, the amount of water drunk during the day, and the amount of coffee or stimulants consumed during the day.

Evaluation of sleep quality was according to Pittsburg Sleep Quality Index (PSQI score).^24^ The level of overactive bladder symptom was evaluated according to Homma's OABSS scale (Table 4).^25^

**Table 4.** Assessment of Overactive Bladder Symptoms using Homma's OABSS Scale

Question	Frequency	Score
How many times do you often urinate from waking in the morning until sleeping at night	≤7	0
	8–14	1
	≥15	2
How many times do you often urinate from sleeping at night until waking in the morning?	0	0
	1	1
	2	2
	≥3	3
How often do you have a sudden desire to urinate, which is difficult to defer?	Not at all	0
	Less than once a week	1
	Once a week or more	2
	About once a day	3
	2–4 times a day	4
	5 times a day or more	5
How often do you leak urine because you cannot defer the sudden desire to urinate?	Not at all	0
	Less than once a week	1
	Once a week or more	2
	About once a day	3
	2–4 times a day	4
	5 times a day or more	5
Evaluation the OAB symptoms levels Mild: ≤5 points Moderate: 6–11 points Severe: ≥12 points		

OAB, overactive bladder.

### Evaluation of undesirable effects of INK treatment

Abnormal paraclinical effects were monitored in symptoms, including but not limited to gastrointestinal adverse effects such as nausea, vomiting, abdominal pain, gastrointestinal disturbances, and diarrhea/constipation; allergic reactions such as allergies and rashes; vital indicators that includes pulse, blood pressure, and respiration rate; and central nervous system (headache, dizziness, vertigo, fatigue). Paraclinical parameters were recorded at the beginning of the study (D0) and after 30 days of treatment (D30), complete blood profiles were performed, including blood counts and biochemical tests, such as AST, ALT, creatinine, and albumin. Ultrasound imaging assessment was performed to evaluate the residual urine in the bladder.

### Data processing

Data were processed by biomedical statistical methods with SPSS 20.0 software for Windows. Statistical analysis was conducted by comparing each study outcome using a paired Student's *t*-test and ratios by square test χ^2^ for a significance level of *P* < .05.

### Research ethics

Before the study, the patients were consulted with medical doctors, and they agreed and signed in a consent form to participate in the study. The study was approved by the Scientific Council of the Hanoi Hospital of Traditional Medicine and the Ethics Council of the Vietnam Institute of Dietary Supplements.

### Research location

The study was performed at the Geriatric Department Outpatient Service of the Hanoi Hospital of Traditional Medicine.

## RESULTS

The average frequency of nocturia, the daily frequency of urination, the number of urinary incontinence in a week, and the number of urgent urination during the day were decreased compared with the levels before treatment. The difference before and after treatment was a statistical significance of *P* < .05. After 30 days of INK phytotherapy, the average number of nocturia was reduced from 4.06 ± 1.53 to 1.14 ± 0.94; the difference was highly statistically significant with *P* < .01 (Table 5).

**Table 5.** Changes in the Urinary Index Before and After Treatment

Index	Before treatment	After treatment	*P*
	(X ± SD)	(X ± SD)	
Average frequency of nocturia (times)	4.06 ± 1.53	1.14 ± 0.94	<.01
Weekly average incontinence of urination (times)	0.92 ± 1.56	0.60 ± 1.0 2	<.05
Daily average urgency of urination (times)	7.67 ± 5.00	5.82 ± 3. 70	<.05
Daily average frequency of urination (times)	9.96 ± 4.04	8.00 ± 3.70	<.05

**FIG. 1.**
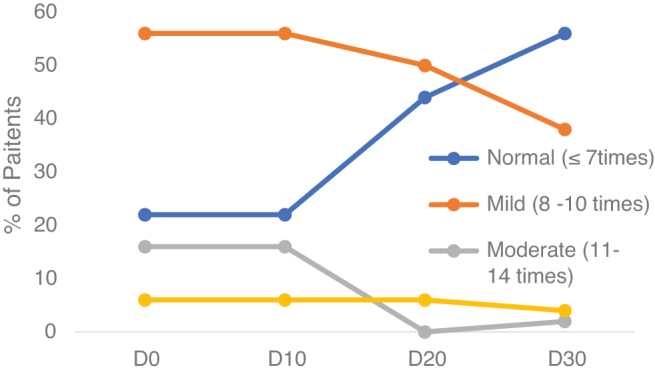
Changes in the level of frequent urination during treatment.

Figure 1 showed at the initial time (D0), patients with a frequency of urination at a mild level (8–10 times) accounted for a highest ratio of 56%, followed by a moderate level (11–14 times) accounting for 16%, and severe (≥15 times) with a low ratio of 6% with three patients. Over the time of treatment, the level of frequency of urination has markedly decreased. At the time of D10, 28% of patients returned to normal; by the time of D20, it was 44%; and at the time of D30, it was 56% (Fig. 1).

Before treatment at D0, severe nocturia (≥3 times) accounted for the highest ratio of 92.0%, moderate level accounted for 4%, and mild level accounted for 4%. At the time of D10, the level of nocturia improved and at the time of D20, the level of nocturia improved markedly: the severity decreased to 32%, and there were 6% no longer nocturia. By the time of D30, the level of nocturia improved markedly, 24% of patients did not urinate at night, and the severity of nocturia was only 10% (Table 6).

**Table 6.** Changes in the Level of Nocturia During Treatment

Time level	D0		D10		D20		D30	
	*n*	%	*n*	%	*N*	%	*N*	%
No peeing at night					3	6.0	12	24.0
Mild (1 time)	2	4.0	2	4.0	17	34.0	25	50.0
Moderate (2 times)	2	4.0	4	8.0	14	28.0	8	16.0
Severe (≥3 times)	46	92.0	44	88 .0	16	32.0	5	10.0
Average frequency of nocturia (times) (X ± SD)	4.06 ± 1.53		3.61 ± 1.22		2.06 ± 1.41		1.14 ± 0.94	
*P*					*P* D20-D0 < 0.03		*P* D30-D0 < 0.01	

**FIG. 2.**
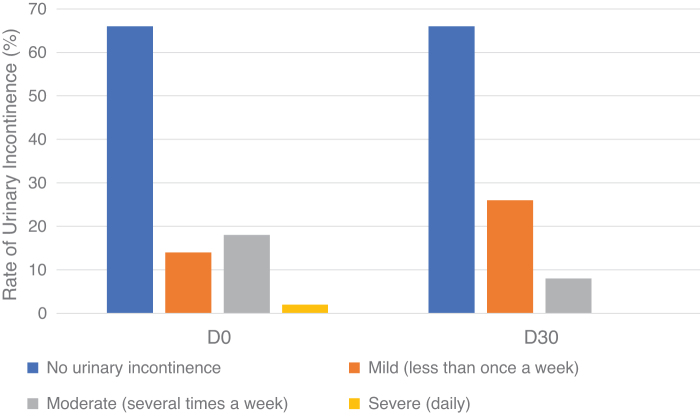
Changes in the level of urinary incontinence during the course of treatment.

Most of the patients at the time before the study did not have symptoms of urinary incontinence with a ratio of 66%, followed by mild incontinence with 14%, moderate level with 18%, and severe level with 2%. After 30 days of treatment, the ratio of patients with no urinary incontinence was 66%, mild urinary incontinence was 26%, the moderate level was 8%, and severe level was 0% (Fig. 2).

The average OABSS score decreased from 9.31 ± 1.44 to 6.8 ± 2.21. The improvement was statistically significant with *P* < .05 (Table 7).

**Table 7.** Characteristics of Homma's OABSS Score Improvement Before and After Treatment

Point	Score TB(X ± SD)		*P*
	D0	D30	
OABSS score (X ± SD)	9.31 ± 1.4 4	6.8 ± 2.21	<.05

After 30 days of treatment, the indicators of sleep quality, sleep time, and performance have all improved. The total PSQI score before treatment was 13.11 ± 1.33, and after treatment decreased to 10.54 ± 2.21. The improvement in sleep was statistically significant, with *P* < .05 (Table 8).

**Table 8.** Characteristics of Improving Sleep Quality According to Pittsburg Sleep Quality Index Score

Time index	D0(X ± SD)	D30(X ± SD)	*P*
Quality of sleep	2.52 ± 0.51	1.93 ± 0.73	<.05
Time to sleep	2.75 ± 0.44	2.26 ± 0.48	
Sleep duration	2.93 ± 0.25	2.36 ± 0.57	
Sleep performance	2.95 ± 0.21	2. 58 ± 0.72	
Disturbance in the night	1.23 ± 0.42	0.85 ± 0.44	
Disturbance of the day	0.73 ± 0.76	0.56 ± 0.49	
Total PSQI	13.11 ± 1.33	10.54 ± 2.21	

PSQI, Pittsburg Sleep Quality Index.

The systolic blood pressure, diastolic blood pressure, and pulse rate before and after treatment were not statistically different with a *P* > .05 (Table 9).

**Table 9.** Changes in Blood Pressure and Pulse Rate Before and After Treatment

Index	D0	D30	*P*
Systolic blood pressure (X ± SD)	119.20 ± 12.71	117.73 ± 10.59	>.05
Diastolic blood pressure (X ± SD)	72.61 ± 8.79	73.36 ± 8.93	
Pulse Frequency: (X ± SD)	84.27 ± 9.10	84.05 ± 7.6	

After 30 days of treatment, the body weight index of the patients changed but there was no statistical significance (*P* > .05; Table 10).

**Table 10.** Change in body weight index (BMI) before and after treatment

Level	D0 (X ± SD)	D30 (X ± SD)	*P*
Thin	16.21 ± 1.34	16.30 ± 1.68	>.05
Normal	20.89 ± 1.10	20.57 ± 1.32	
Fat	25.04 ± 2.51	24.97 ± 2.32	
Average BMI (X ± S)	21.71 ± 2.7 7	21.63 ± 2.64	

### Assessment of the paraclinical results

The changes in the mean value of red blood cell count, hemoglobin value, white blood cell count, and platelet count after 30 days of treatment compared with before treatment levels were not statistically significant with *P* > .05 (Table 11).

**Table 11.** Changes in Some Hematological Indices Before and After Treatment

Time index	D0(X ± SD)	D30 (X ± SD)	*P*
Red blood cell count (10^12^/L)	4.53 ± 0.34	4.47 ± 0.30	>.05
Hemoglobin (g/dL)	126.61 ± 6.70	127.36 ± 6.02	
WBC count (10^9^/L)	6.31 ± 1. 01	6.27 ± 0.63	
Platelet count (10^9^/L)	245.43 ± 44. 57	252.73 ± 40. 95	

The changes in mean values of albumin, creatinine, AST, and ALT after 30 days of treatment compared with before treatment were statistically insignificant, with *P* > .05 (Table 12).

**Table 12.** Changes in Blood Biochemical Indices Before and After Treatment

Time index	D0(X ± SD)	D30(X ± SD)	*P*_0–30_
Albumin (g/L)	41.80 ± 3.26	41.86 ± 3.91	>.05
Creatinine (μmol/L)	79.35 ± 13.61	79.73 ± 12.91	
AST (U/L)	33.37 ± 11.88	33.37 ± 10.10	
ALT (U/L)	29.41 ± 13.46	30.59 ± 12.74	

There was a tendency of improvement in bladder evacuation of residual urine after 30 days of INK treatment with residual urine decreased from 21.22 ± 22.21 to 18.12 ± 17.30 (mL); however, the difference was not statistically significant with *P* > .05 (Table 13).

**Table 13.** Change in Residual Urine Before and After Treatment

Time	Volume TB(X ± SD)		*P*
	D0	D30	
Mean amount of residual urine (mL)	21.22 ± 22.21	18.12 ± 17.30	>.05

In addition to the above paraclinical investigation, all patients were tested for urinalysis before and after treatment in this study. The results showed that the pH, protein, glucose, nitrite, ketone, bilirubin, urobilinogen, white blood cell, and red blood cell counts were within normal ranges.

### Monitoring subjective side effects

All 50 patients completed the study, and no patient had symptoms related to intolerance or allergic reactions to the INK treatment.

## DISCUSSION

OAB can have a significant negative impact on just about every aspect of a patient's life. Despite extensive research and treatment spending, conventional medicine has not developed a safe and adequately effective treatment for OAB that plagues many people, especially aging women and men.

Pharmaceutical drugs commonly used for OAB are expensive and side effect prone. Only a small proportion of the affected population can get some benefit from continuing with the conventional treatment for OAB. Fortunately, numerous traditional and natural therapies in all parts of the world have been used safely and effectively to control urination disorders and symptoms related to OAB.

Our current study evaluated the effectiveness and safety of INK phytotherapy for 50 patients with typical symptoms and life- and health-related problems of OAB. INK was formulated by a combination of the traditional herbal agent *Eucommia ulmoides*, which is empirically used for urination disorders and neurological imbalance, and L-carnitine fumarate, a nutritional agent that supports hormonal balance and energy metabolism. Additionally, INK included Go-Less, a proprietary blend of a water-soluble pumpkin seed extract (EFLA 940), and the soy germ extract, which has been shown to improve the symptoms of OAB in several clinical studies.^26–29^

The results of our study demonstrated remarkable clinical benefits of INK for control and improvement of all symptoms and quality-of-life parameters in patients with OAB. After 30 days of treatment, all functional symptoms improved: the average frequency of nocturia decreased from 4.06 ± 1.53 to 1.14 ± 0.94 (mean decrease of 2.92 times); the daily average frequency of urination decreased from 9.96 ± 4.04 to 8.00 ± 3.70; the daily average urgency of urination decreased from 7.67 ± 5.00 to 5.82 ± 3.7. The improvement of major OBA urinary symptoms before and after 30 days of INK treatment was statistically significant with *P* < .05.

INK treatment significantly reduced the average frequency of nocturia, which benefited OAB patients tremendously. In addition to sleep interruption, resulting fatigue, and poor quality of life, nocturia could increase the risks of falls and fractures, which are associated with high mortality in elderly patients. Approximately 33% of older adults do not survive beyond 1 year after a hip fracture.^30^

Homma's OABSS is a well-defined, easy-to-understand questionnaire. OABSS has high sensitivity in the diagnosis and evaluation of treatment effectiveness of OAB treatment. The results of the current study showed that the average OABSS score, according to Homma's OABSS decreased from 9.31 ± 1.44 to 6.8 ± 2.2 1. The improvement of symptoms before and after treatment was statistically significant with *P* < .05. These data strongly indicated INK effectiveness for controlling OAB symptoms and improving the patient's quality of life.

Sleep disturbance and fatigue are present in substantial percentages of patients with OAB. Among patients with OAB, sleep disturbance and fatigue were associated with more severe OAB symptoms, worse health-related quality of life, and poorer psychosocial health.^31^ Around 96% of the patients in our study had poor sleep quality mostly due to having frequent nocturia with three to five times waking up at night to urinate. The assessment of sleep before and after treatment found that the improvement in sleep was expressed in the time to fall asleep and the duration of sleep. After 30 days of using INK, all sleep quality indicators improved with statistical significance (*P* < .05) with total PSQI score decreasing from 13.11 ± 1.33 to 10.54 ± 2.21. The above improvement in sleep quality was due mainly to the patient's reduction in nocturnal urination.

There were tendency and statistically significant improvements in OAB symptom scores during the progress of the study. All the indicators were remarkably improved after 20 days and 30 days of the treatment with INK. This indicated that a longer than 30 days' course of treatment might produce even better clinical benefits for the patients. Additionally, there was a tendency of improvement in bladder evacuation after 30 days of INK treatment with residual urine decreased from 21.22 ± 22.21 to 18.12 ± 17.30 (mL). This suggests that a longer course of treatment might restore urine bladder function that would positively contribute to the long-term OAB control and reduction of urination infection risks for the patients

It has been observed that OAB symptoms are significantly higher and more frequently observed in women than in men, especially OAB with stress urinary incontinence and nocturia.^32–34^

In this study, the women accounted for 94.0% of studied patients. We believe that in male subjects, prostate gland enlargement is the leading cause of lower urinary tract symptoms and that an OAB is considered secondary. Because the study subjects included patients only with primary OAB, we excluded the majority of males with OAB symptoms because of associated prostate pathology.

The studies of OAB symptoms have historically focused on women. However, there was evidence that men, including those with benign prostate hypertrophy, have OAB symptoms that are usually untreated. In one of the studies, a population of 7,244,501 patients ≥45 years with an OAB diagnosis, 24.4% were treated; 75.6% went untreated. Only 25.6% of those treated were men. More importantly, there was a significantly (*P* < .001) decreased proportion of men treated compared with women in every age group.^35^ The evidence of the usefulness of INK suggested that it should be investigated and applied to men with OAB symptoms, even with associated prostate disorders.

The current study also revealed a remarkable superiority of INK that showed no side effects and complications in subjective complaints, medical observation, and objective paraclinical investigation. That suggests INK might be used as long as needed or as maintenance therapy for patients with refractory and recurrent OAB.

INK's effectiveness and excellent safety profile in our study suggest that the studies with larger samples and control groups for a long time are encouraged to promote a more comprehensive application of this potentially useful natural product for patients with OAB and other age-related urinary disorders.

INK food supplement is effective and safe for patients with primary OAB symptoms in 30 days of treatment. More extensive control clinical trials are warranted to prove our findings and promote wider use of INK for OAB and possible other age-related urination disorders.
